# Analysis of influencing factors and construction of prediction model for postoperative nausea and vomiting in patients undergoing laparoscopic sleeve gastrectomy: a single-center retrospective cohort study

**DOI:** 10.1186/s12871-024-02502-z

**Published:** 2024-04-05

**Authors:** Bucheng Liao, Wuhao Liao, Xinhai Wu, Shujuan Liu, Yanze Li, Ruixia Qin, Shuang Yin

**Affiliations:** 1 Department of Anesthesiology, Peking University Shenzhen Hospital, No. 1120, Lianhua Street, Shenzhen, 518000 Guangdong China; 2 Department of Anesthesiology, Shenzhen Hospital of Southern Medical University, No. 1333, Xinhu Street, Shenzhen, 518000 Guangdong China

**Keywords:** Laparoscopic sleeve gastrectomy, Postoperative nausea and vomiting, Multivariate analysis, Nomogram, Prediction model

## Abstract

**Background:**

With the increasing number of bariatric surgeries, the high incidence of postoperative nausea and vomiting (PONV) associated with this surgery has also gradually attracted attention. Among the common bariatric surgery methods, patients undergoing sleeve gastrectomy (SG) have the highest incidence of nausea and vomiting. The mechanism of occurrence of PONV is very complex. This study aims to explore the influencing factors of PONV in patients undergoing laparoscopic sleeve gastrectomy (LSG) and construct a nomogram prediction model based on these factors.

**Methods:**

With the approval of the Ethics Committee, the electronic medical records of patients who underwent LSG from July 2022 to May 2023 were collected retrospectively.

**Results:**

A total of 114 patients with complete medical records who underwent LSG from July 2022 to May 2023 were included in this study. Among them, 46 patients developed PONV, resulting in a PONV incidence rate of 40.4%. Multivariate logistic regression analysis revealed that female gender, the use of inhalation anesthesia, and operation time ≥ 120 min were risk factors for PONV in LSG. Additionally, the use of more than two kinds of antiemetic drugs was identified as a protective factor. Based on these factors, a nomogram model was constructed.

**Conclusion:**

PONV in patients undergoing LSG is related to gender, type of anesthesia, duration of surgery, and combination therapy with antiemetic drugs. The nomogram prediction model constructed in this study demonstrates high accuracy and discrimination in predicting the occurrence of PONV in patients undergoing LSG.

## Introduction

At present, approximately 600 million adults worldwide are obese [1], with the incidence rate of overweight and obesity among Chinese adults standing at about 50.7% [2]. Bariatric surgery is the most effective method for treating obesity, with sleeve gastrectomy (SG) emerging as the fastest-growing bariatric surgery and accounting for the largest number of cases in recent years. According to a 2014 survey conducted by the International Federation for the Surgery of Obesity and Metabolic Disorders (IFSO), SG represented 45.9% of all bariatric surgeries, ranking first [3]. Concurrently, the high incidence of postoperative nausea and vomiting (PONV) among these patients has garnered increasing attention. Relevant research findings indicate that PONV in laparoscopic sleeve gastrectomy (LSG) patients can reach 58.6% [4], significantly surpassing the overall incidence of PONV in surgical patients by 30% [5].

PONV not only significantly reduces patient satisfaction [6, 7] but also increases the incidence of postoperative complications, such as anastomotic leakage, incisional hernia formation, and gastroesophageal reflux [8]. Additionally, it prolongs patient hospitalization time [9] and increases the medical expenses borne by patients [10].

Many previous studies have elucidated the influencing factors of PONV in traditional surgery [11], and measures taken to address these factors can, to some extent, prevent the occurrence of PONV. However, there is a lack of research on the influencing factors of PONV in laparoscopic sleeve gastrectomy (LSG). This study explores the influencing factors of PONV in patients undergoing LSG and constructs a nomogram prediction model based on them.

## Materials and methods

### Study design

After the approval of the IRB Ethics Committee, the records of patients who underwent LSG between July 2022 and May 2023 were retrospectively reviewed, and the requirement for written informed consent was waived due to the retrospective nature of the study.

The inclusion criteria include patients aged 18 to 65 who underwent laparoscopic sleeve gastrectomy (LSG) under general anesthesia at Peking University Shenzhen Hospital between July 2022 and May 2023, with a surgical duration of 60 min or more.

The exclusion criteria include unsuccessful completion of the surgery and missing PONV data (such as postoperative death/continuous sedation in the ICU (Intensive care unit), which cannot be evaluated for the presence of PONV).

### Definition of outcome variables

PONV is defined as any recorded nausea and/or vomiting event that occurs during PACU treatment or in a surgical ward. Additionally, we conducted follow-up assessments for PONV within 24 h after surgery.

### Anesthetic protocol

Propofol at a dosage of 2-2.5 mg/kg, sufentanil at a dosage of 0.4–0.6 µg/kg, and either rocuronium at a dosage of 0.6 mg/kg or atracurium at a dosage of 0.5–0.6 mg/kg were used for anesthesia induction. Tracheal intubation was performed three minutes later for mechanical ventilation. The tidal volume (TV) was set at 6–8 ml/kg, and the respiratory rate was adjusted based on the end-expiratory carbon dioxide level to maintain it at 35–45 mmHg.

During the intraoperative anesthesia management, sevoflurane or propofol was used to maintain sedation, remifentanil was administered to ensure analgesic effect, and rocuronium or atracurium was intermittently injected during surgery to ensure muscle relaxation. The goal was to maintain the BIS value between 40 and 60 or keep the baseline blood pressure fluctuation within 20%. In case of hemodynamic fluctuations during surgery, vasoactive drugs or infusions would be administered based on the specific situation.

In our hospital, antiemetic drugs such as dexamethasone at a dosage of 5 mg, azasetron at a dosage of 10 mg, and droperidol at a dosage of 1 mg are used. Anesthesiologists can choose to use one or more of these drugs in combination. After the surgery, the patient is transferred to the postanesthesia care unit (PACU) to have the tracheal catheter removed. Once the exit criteria are met, the patient is transferred back to the ward.

Patient-controlled analgesia (PCA) after surgery is determined by the patients themselves. The primary analgesic drug used for PCA is sufentanil. The medical ward has the discretion to administer additional analgesics based on the patient’s postoperative pain, which may include nonsteroidal anti-inflammatory drugs (NSAIDs) like flurbiprofen, morphine, and others.

### Data acquisition

The patients who were selected were divided into two groups: the PONV group and the non-PONV group, based on whether they experienced PONV after LSG. The occurrence of PONV was determined by reviewing the electronic medical record system, considering the patient’s experience of PONV within 24 h of postoperative recovery as the dependent variable. If nausea and vomiting were recorded in the medical record within 24 h of surgical recovery, it was considered that the patient had PONV.

Based on the known influencing factors for PONV [12, 13], we consider factors that can directly or indirectly affect PONV as independent variables. We collect preoperative data, intraoperative data, and postoperative data separately, as outlined below:

Preoperative data is gathered through an electronic medical record system, including age, gender, comorbidities, American Society of Anesthesiologists (ASA) Physical Status, and more.

Intraoperative data is obtained from the electronic anesthesia recording system, which includes information such as the type of anesthesia, blood pressure, heart rate, intraoperative medication, duration of surgery, and blood transfusion or infusion. The duration of surgery refers to the time from the beginning to the end of the surgery, expressed in minutes. The intraoperative infusion or blood transfusion volume is standardized by body weight and expressed in ml/kg.

Postoperative data is obtained by reviewing the electronic medical record system. This data includes the occurrence of PONV, whether patient-controlled analgesia (PCA) was used, and whether there were any hospital deaths. The drug sufentanil, which is included in PCA, is an opioid drug. Therefore, its use will be considered in the postoperative statistics. Additionally, we will determine whether opioid drugs were used for remedial analgesia in the ward based on electronic medical records.

The interval between intraoperative blood pressure and heart rate recordings is 5 min for recording and counting the cumulative time of hemodynamic fluctuations. The accumulated time of intraoperative hypotension, hypertension, tachycardia, or bradycardia is the sum of the times when these conditions occur during the operation. Intraoperative blood pressure is mainly measured non-invasively through arterial pressure, which can be replaced by invasive arterial pressure when performing invasive arterial monitoring. Some operations, such as arterial blood extraction, can cause false fluctuations in blood pressure. The criteria for excluding such data are as follows: (1) Systolic blood pressure (SBP) ≤ 20 mmHg or ≥ 300 mmHg. (2) SBP change ≥ 80 mmHg/min.

Intraoperative hypotension is defined as SBP < 90 mmHg, and intraoperative hypertension is defined as SBP ≥ 160 mmHg. Intraoperative tachycardia is defined as a heart rate > 100 beats/min, and intraoperative bradycardia is defined as a heart rate < 50 beats/min. When the accumulated time of intraoperative hypotension, hypertension, tachycardia, or bradycardia is ≥ 5 min, it will be recorded as such during the operation.

### Statistical analysis

SPSS 23.0 statistical software was used for data processing. The measurement data conforming to a normal distribution were expressed as mean ± standard deviation (x ± s), and an independent sample t-test was adopted. The measurement data of a non-normal distribution were expressed by the median (interquartile interval) [M (Q1, Q3)], and the Mann-Whitney U test was used. The counting data were described in terms of examples and percentages (%), using χ^2^ inspection.

Factors with significant differences in PONV occurrence in single-factor analysis were selected for multivariate logistic regression analysis to screen out PONV-related influencing factors. In the above analysis, *P* < 0.05 was taken to indicate statistical significance.

R software was used to draw a nomogram prediction model for the selected PONV influencing factors. By drawing the receiver operating characteristic curve (ROC curve), the area under the ROC curve (AUC) was calculated to evaluate the accuracy of the prediction model. Calibration curves were drawn to evaluate the consistency of the nomogram prediction model. The Hosmer-Lemeshow test was used to evaluate the goodness of fit of the model, with *P* > 0.05 indicating good goodness of fit.

## Results

**Fig. 1:**
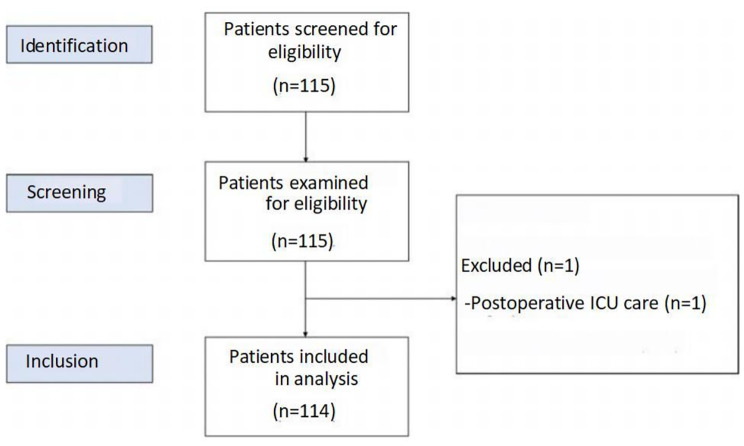
Flow chart of cohort selection ICU, Intensive care unit

A total of 115 patients aged 18 to 65 underwent LSG under general anesthesia, with a duration of more than 60 min. A patient was excluded from the analysis as they were transferred to the ICU after surgery (Fig. 1).

In this study, 46 out of 114 LSG patients (40.4%) developed PONV. The patients were grouped based on the occurrence of PONV, and it was found that there was no statistically significant difference in preoperative basic information between the two groups, except for gender (*P* < 0.05). During surgery, a higher number of patients in the PONV group used inhalation anesthesia, had a surgery time of ≥ 120 min, and did not receive combined treatment with antiemetic drugs (*P* < 0.05). There was no significant difference in the postoperative use of opioid drugs between the two groups (*P* > 0.05). The potential influencing factors and occurrence of PONV are shown in Table 1.

**Table 1:** Potential influencing factors and occurrence of PONV

Potential influencing factor	PONV	Non-PONV	*P* value
No. of patients	46	68	
**Preoperative**			
Age (years)	31.1 ± 7.1	33.8 ± 7.5	0.056
Sex(Male/Female)	7/39	26/42	0.012
Smoking history(yes/no)	4/42	11/57	0.246
BMI(kg/m^2^)	36.2(32.8, 39)	36.4(33, 39)	0.171
ASA Physical Status(II/III)	35/11	41/27	0.079
Comorbidity			
-Hypertension(yes/no)	32/14	52/16	0.411
-Diabetes(yes/no)	33/13	50/18	0.833
-Obstructive sleep apnea syndrome (yes/no)	7/39	17/51	0.209
-Arrhythmias(yes/no)	0/46	4/64	0.094
-Anemia(yes/no)	0/46	2/66	0.241
**Intraoperative**			
Duration of surgery ≥ 120 min(yes/no)	23/23	20/48	0.026
Infusion volume(ml/kg)	13.1(10.9, 14.9)	12.8(10.3, 15.2)	0.797
Type of anesthesia (Inhalation anesthesia/Total intravenous anesthesia)	41/5	49/19	0.028
Intraoperative hemodynamic fluctuations			
-Hypotension(yes/no)	6/40	8/60	0.838
-Hypertension(yes/no)	8/38	20/48	0.144
-Tachycardia(yes/no)	2/44	2/66	0.689
-Bradycardia(yes/no)	5/41	9/59	0.706
Types of antiemetic drugs(None/One drug/Two or more drugs)	0/22/24	0/20/48	0.046
Combined with anticholinergic drugs(yes/no)	8/38	16/52	0.430
Combined with dexmedetomidine(yes/no)	20/26	29/39	0.930
**Postoperative**			
Combined with opioid drugs(yes/no)	20/26	38/30	0.194

Normal distribution of data were expressed as mean ± standard deviation. Non-normal distribution of data were expressed by the median (interquartile interval). PONV, Postoperative nausea and vomiting; BMI, Body mass index; ASA, American society of aneshesiologists; OSAS, Obstructive sleep apnea syndrome

We conducted a multivariate logistic regression analysis on the statistically significant factors mentioned above. The analysis revealed that gender (odds ratio (OR) = 4.452, *P* = 0.006), inhalation anesthesia (OR = 3.877, *P* = 0.032), surgical time ≥ 120 min (OR = 2.973, *P* = 0.016), and the combination of two or more antiemetic drugs acted as protective factors for PONV (OR = 0.312, *P* = 0.012). The results of the multivariate logistic regression analysis for PONV are presented in Table 2.

**Table 2:** Multivariate logistic regression analysis of PONV

Influencing factors	Β value	SE	Wald value	*P* value	Adjusted OR	95%CI
Female	1.493	0.546	7.470	0.006	4.452	1.526 ~ 12.99
Inhalation anesthesia	1.355	0.630	4.623	0.032	3.877	1.127 ~ 13.332
Duration of surgery ≥ 120 min	1.090	0.451	5.849	0.016	2.973	1.299 ~ 7.189
Antiemetic drugs combination therapy	-1.165	0.465	6.269	0.012	0.312	1.288 ~ 7.981
Constant	-0.447	0.413	1.173	0.279	0.640	

PONV, Postoperative nausea and vomiting; OR, odds ratio; CI, confidence interval

**Fig. 2:**
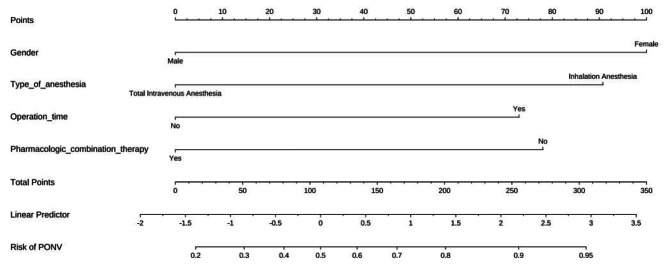
Prediction model of PONV nomogram in patients undergoing LSG. PONV, Postoperative nausea and vomiting.

**Fig. 3:**
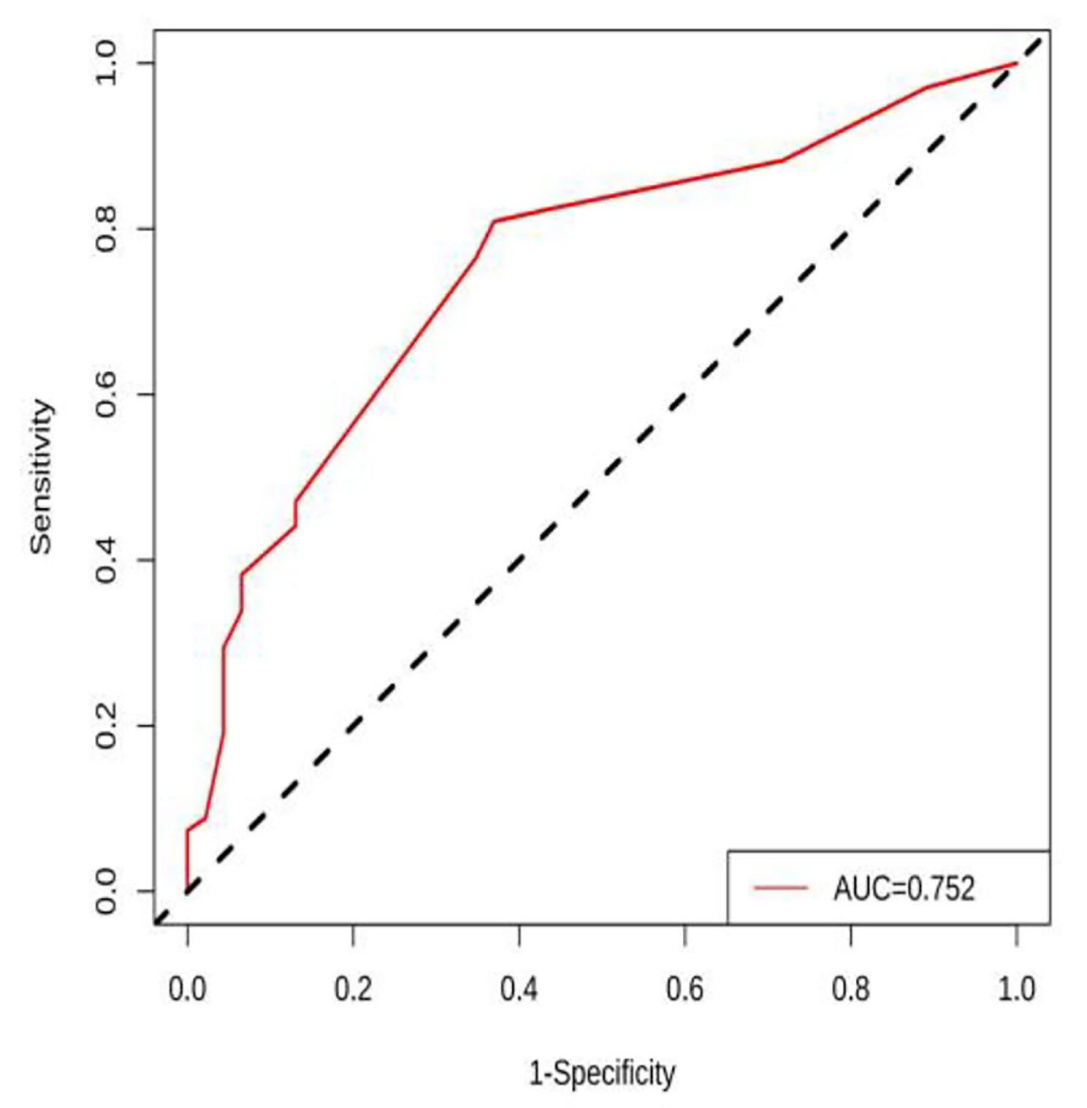
ROC curve

**Fig. 4:**
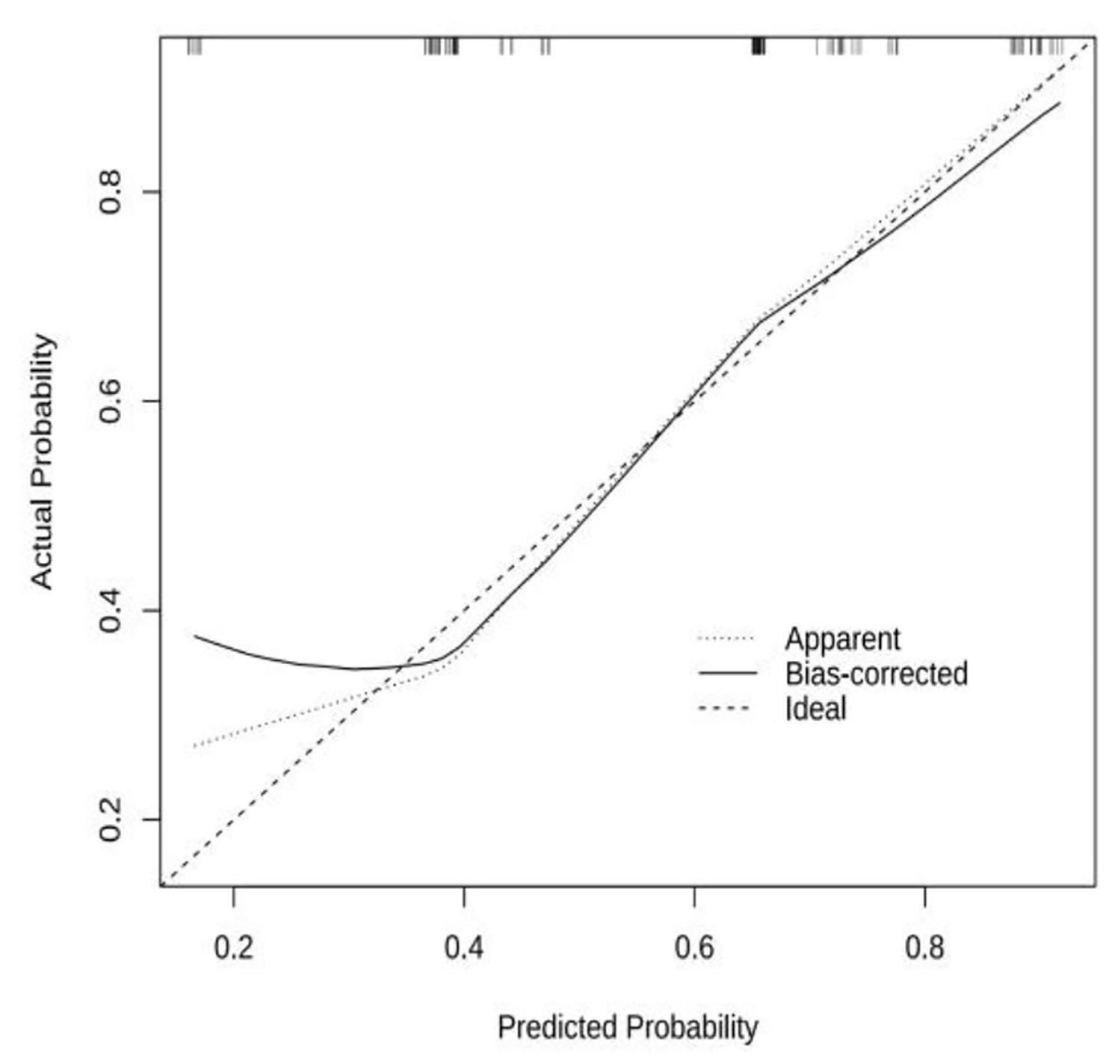
Calibration curve

According to the independent influencing factors identified in the multivariate analysis, a function for the factors influencing PONV in patients undergoing LSG was constructed. The model expression is as follows: *P* = 1/[1 + e^−(−0.447+1.493×Gender+1.355×Type of anesthesia+1.090×Duration of surgery−1.165×Antiemetic drugs combination therapy)^]. This model was created using the R software component line graph model (Fig. 2). The prediction model of the PONV nomogram in patients undergoing LSG is shown in Fig. 2. The predictive value of the nomogram model was evaluated using the ROC analysis method, with an area under the curve of 0.752 and a 95% CI of 0.662–0.843. These results indicate that the model established in this study has good discrimination. The ROC curve is displayed in Fig. 3. Next, the nomogram was internally verified using the bootstrapping method, and the calibration curve was established by repeated sampling the original data 1000 times. The calibration curve is shown in Fig. 4. It can be observed from the figure that the predicted probability of occurrence aligns well with the actual probability of occurrence. The Hosmer-Lemeshow test was performed, yielding X^2^ = 3.558 and *P* = 0.736. These results indicate a good fit between the predicted model and the observed values.

## Discussion

This study demonstrates that the incidence of PONV within 24 h of recovery after laparoscopic sleeve gastrectomy (LSG) is 40.4% (46 cases). Women, inhalation anesthesia, and a surgery duration of ≥ 120 min were identified as independent risk factors for PONV. Additionally, the use of a combination of two or more antiemetic drugs was found to be a protective factor against PONV.

Apfel et al. [11] conducted a meta-analysis of 22 studies on risk factors for PONV. The results indicated that patients’ individual risk factors ranked as follows in terms of their contribution: being female (OR = 2.57, 95% CI 2.32–2.84), having a history of PONV (OR = 2.09, 95% CI 1.90–2.29), being a non-smoker (OR = 1.82, 95% CI 1.68–1.98), having a history of motion sickness (OR = 1.77, 95% CI 1.55–2.04), and experiencing a 10-year increase in age (OR = 0.88, 95% CI 0.84–0.92). This study found that women are at a higher risk of PONV after LSG, which is consistent with the research findings of Apfel et al. [11]. This is primarily attributed to the disparity in hormone levels between men and women, particularly the elevated levels of serum sex hormones, progesterone, and estrogen in female patients. Hormonal fluctuations post-surgery are substantial, leading to symptoms such as nausea, vomiting, and irritability in some female patients [14].

However, unlike the findings of Apfel et al. [11], this study discovered that a smoking history was not a risk factor for PONV after LSG. This discrepancy may be due to the small proportion of smokers in this study, which prevented the impact of tobacco on reducing the occurrence of PONV from being reflected. Nonetheless, it should be noted that nicotine, polycyclic aromatic hydrocarbons, and other substances in tobacco can diminish nerve receptor function, induce an increase in Cytochrome P450 Isozyme expression, and enhance the body’s tolerance to surgery and narcotics, thereby reducing the likelihood of PONV [15].

Furthermore, all patients included in this study were middle-aged and young, and the age difference between the PONV group and the non-PONV group was similar. Consequently, no correlation between age and PONV was observed in this study.

The results of this study showed a significant increase in the incidence of PONV in patients with a surgery time ≥ 120 min, which is consistent with the results of Apfel et al. [11]. The reason may be related to the use of drugs during the operation. As the operation time extends, the amount of sedatives, analgesics, and muscle relaxants used during the operation will increase accordingly. The accumulation of drugs in the body stimulates the mucosa of the digestive tract, causing the intestinal Chromaffin cells to release neurotransmitters that stimulate the Vagus nerve and visceral nerve afferent fibers in the intestinal wall. This transmission of stimulus signals to the vomiting center or initiation of the vomiting reflex occurs through the chemoreceptor trigger area.

Additionally, relevant studies [4] have shown that for every 30-minute increase in surgical duration, the incidence of PONV increases by 60%. This increase may be attributed to the prolonged use of large amounts of potential emetic drugs. Therefore, reducing the patient’s surgical time through skilled surgical procedures can help mitigate the occurrence of PONV in patients.

The research results indicate that the incidence of PONV is lower in patients receiving total intravenous anesthesia compared to those undergoing inhalation anesthesia, which aligns with the findings of Scheiermann P’s study. The reason for this difference may be attributed to the stimulating effect of inhaled anesthetics on the vomiting center of the cerebral cortex; however, the specific mechanism remains unknown [16]. Furthermore, studies have demonstrated that patients under sevoflurane anesthesia exhibit higher levels of motilin, which is closely associated with nausea and vomiting. It is possible that sevoflurane increases the occurrence of nausea and vomiting by modulating motilin production. On the other hand, propofol possesses certain antiemetic properties, possibly by mediating γ-aminobutyric acid receptors, leading to a decrease in serotonin (5-HT) concentration and inhibiting the chemical receptor vagus nucleus, thereby producing an antiemetic effect [17].

The incidence of PONV in patients undergoing laparoscopic sleeve gastrectomy (LSG) in this study was lower (40.4%) compared to previous studies (58.6%) [4]. This difference can be attributed to the multi-channel prevention of PONV in our hospital, which involves the combined use of antiemetic drugs with different mechanisms (with two or more drugs accounting for 66.7%) [18]. Apart from dexamethasone, our hospital also combines a 5-HT3 receptor antagonist (azasetron) and/or butyrophenone drugs (droperidol) simultaneously when using more than two types of anti-vomiting medications to achieve a favorable clinical effect of prevention. This finding aligns with the conclusions of Zaina Naeem et al. [19] and confirms that the combination of multiple drugs, as recommended for high-risk PONV patients, is equally effective for patients undergoing LSG.

Furthermore, some studies [20] have suggested that high-risk patients should consider supplementing with a third, and possibly a fourth, type of antiemetic drug with different mechanisms. Whether the routine application of three or more antiemetic drugs to bariatric surgery patients can yield more effective results requires further investigation. Additionally, the potential side effects of combined medications should also be taken into consideration. Overall, adverse events associated with antiemetics are relatively rare, and the quality of evidence regarding these events is low. However, when patients experience adverse drug reactions, they should not be ignored. Common side effects may include headache, dizziness, restlessness, sedation, constipation, dry mouth, blurred vision, prolonged QT interval, allergic reactions, extrapyramidal reactions, and other symptoms.

The prediction model established in this study is only applicable to predicting PONV in patients with LSG for more than 1 h, and not applicable to other types of bariatric surgery. The reason is that a survey conducted by P. Ziemann Gimmel et al. revealed that the incidence of PONV varies across different surgical procedures in bariatric surgery [4]. The reported incidences of PONV for each surgical procedure were as follows: SG—58.6%, laparoscopic Roux-en-Y gastric bypass (LRYGB)—19.4%, gastric banding (GB)—0%, revision LRYGB—23.1%, and Conversion—0%. The variation in the incidence of PONV mentioned above may be attributed to the divergent alterations in gastric pressure and compliance induced by distinct surgical techniques [21, 22]. The fundus of the stomach is excised in LSG, resulting in a reduction in the distensibility and compliance of the remaining stomach post-surgery, leading to a significant elevation in gastric pressure after surgery and an increased susceptibility to PONV.

Additionally, it is worth investigating the potential impact of changes in ghrelin levels on PONV. Ghrelin has been shown to possess antiemetic properties and its mechanism of action is associated with alleviating gastric paresis [23]. However, it should be noted that ghrelin primarily originates from X/A-like cells located in the gastric fundus mucosa. Previous studies have demonstrated a decrease in ghrelin levels among patients following LSG [24]. However, the clinical confirmation of whether alterations in ghrelin levels impact the incidence of PONV remains pending.

Due to the characteristics of retrospective studies, this study has several limitations. Firstly, there are fewer patients included in this study, which may have a certain impact on the stability of the logistic regression analysis results and introduce bias in the modeling outcomes. Secondly, the lack of a history of motion sickness/nausea and vomiting and the specific dosage of postoperative opioids may affect the accuracy of the prediction model, and since the operation time of the included cases was more than 1 h, the model is not suitable for rapid LSG. Lastly, this study was conducted at a single center, and the generalizability of the findings requires further validation with multi-center data.

In conclusion, there have been few studies on the prediction model of the PONV nomogram after LSG. This study conducted logistic regression analysis based on single-factor analysis and obtained four independent influencing factors of PONV, which include gender, anesthesia mode, operation time, and anti-vomiting drug combination treatment. Furthermore, a prediction model for the PONV nomogram was established. We believe that this model can provide personalized assessment and clinical suggestions for the development of PONV after LSG. However, it is important to note that since this is a retrospective study, this conclusion still needs to be supported by a multicenter and large-scale randomized controlled trial.

## Data Availability

The datasets used and/or analyzed during the current study are available from the corresponding author on reasonable request.

## Abbreviations

PONV: Postoperative nausea and vomiting
SG: Sleeve gastrectomy
LSG: Laparoscopic sleeve gastrectomy
TV: Tidal volume
EPV: Events per variable
ROC: Receiver operating characteristic
AUC: Area under curve
SBP: Systolic blood pressure
PCA: Patient controlled analgesia
ICU: Intensive care unit
BMI: Body mass index
ASA: American society of aneshesiologists
OSAS: Obstructive sleep apnea syndrome
PACU: Postanesthesia care unit
NASID: Nonsteroidal anti-inflammatory drugs
LRYGB: Laparoscopic Roux-en-Y gastric bypass
GB: Gastric banding
